# Genome-Wide Structural Variation Analysis and Breed Comparison of Local Domestic Ducks in Shandong Province, China

**DOI:** 10.3390/ani14243657

**Published:** 2024-12-18

**Authors:** Pengwei Ren, Meixia Zhang, Muhammad Zahoor Khan, Liu Yang, Yadi Jing, Xiang Liu, Xiaohui Yang, Chaoran Zhang, Min Zhang, Zhiming Zhu, Nenzhu Zheng, Lujiao Zhang, Shuer Zhang, Mingxia Zhu

**Affiliations:** 1 College of Agriculture and Biology, Liaocheng University, Liaocheng 252000, China; 2 Shandong Animal Husbandry Station, Jinan 250010, China; 3 Fujian Key Laboratory of Animal Genetics and Breeding, Institute of Animal Husbandry and Veterinary Medicine of Fujian Academy of Agricultural Sciences, Fuzhou 350013, China; 4 Weihai Wendeng District Animal Husbandry and Veterinary Career Development Center, Weihai 264400, China

**Keywords:** duck, structural variants, selective signal analysis, variety characteristics

## Abstract

Structural variations in the duck genome significantly impact the environmental adaptability and phenotypic diversity of duck populations. Characterizing these SVs in local domestic duck breeds from Shandong province offers valuable insights for breed selection and the development of new breeds. This study aimed to profile the genomic SVs in three local duck breeds (Matahu duck, Weishan partridge duck, and Wendeng black duck) and explore their differential distributions. A total of 21,673 SVs were detected using LUMPY (v0.2.13) and DELLY (v1.0.3) software, with 46% located in intergenic regions, 33% in intronic regions, and frameshift deletions being the most prevalent in exonic regions (3%). SVs distribution showed a decreasing trend with shorter chromosome lengths. Population structure analysis revealed distinct genetic profiles, with Matahu and Weishan partridge ducks showing closer affinities and the Wendeng black duck having a more homogeneous genetic background, likely due to geographic isolation. Functional annotation identified genes related to nervous system development, mitosis, spindle assembly, and energy metabolism. Notable genes included *PLXNA4*, *NRP2*, *SEMA3A*, *PTEN*, *MYBL2*, *ADK*, and *COX4I1*. Additionally, genes such as *PRKG1*, *GABRA2*, and *FSHR* were linked to energy metabolism and reproductive activity. The study provides a comprehensive analysis of SVs, revealing significant genetic differentiation and identifying genes associated with economically important traits, offering valuable resources for the genetic improvement and breeding of local duck breeds.

## Simple Summary

The duck industry is an important pillar of China’s livestock industry; however, the development of China’s duck industry has been constrained by the low productivity of local domestic duck breeds due to unsystematic selection and breeding. Matahu duck, Weishan partridge duck, and Wendeng black duck are endemic breeds in Shandong with complex genetic backgrounds, which are natural material pools for duck selection and improvement, but the structural variation data at the genomic level have not yet been resolved and are still to be mined. In this study, we synthesized the SV datasets of three breeds using two software programs, LUMPY and DELLY, and counted the distribution of SVs. On this basis, we analyzed their population genetic structure and preliminarily inferred the kinship relationship of the three breeds. This is consistent with the results of our previous research based on single nucleotide polymorphism loci. We used a selection signal analysis method (Fst) for any two of the three breeds among the varieties. We found a significant enrichment of GO entries and KEGG pathways regarding nervous system development in the different breeds. In addition, some genes related to spindle assembly and energy metabolism were also mined. This study identified and annotated the structural variation of three local domestic duck breeds, preliminarily deduced the affinities of the local domestic duck breeds, and divided the genetic differences among different breeds, which provides an important reference for the conservation of duck breed resources and the cultivation of new breeds in China.

## 1. Introduction

Structural variants (SVs) are defined as DNA sequence variations greater than 50 base pairs in length. The main types of SVs include insertions, deletions, duplications, inversions, and ectopic variations, which can affect larger genomic regions than single-nucleotide variants or short insertions and deletions [1]. When structural variations occur, they can lead to alterations in gene dosage, disrupt gene function, and expose recessive alleles, thereby influencing gene expression regulation, transcription, and translation. Consequently, identifying and characterizing SVs is crucial for understanding the genetic variation associated with diverse traits [2].

Structural variations (SVs) play a critical role in shaping economically significant traits, morphological features, disease resistance, and evolutionary adaptations across diverse animal species, as demonstrated in numerous studies [3,4,5]. In the context of poultry research, Rice et al. advanced the field by classifying and resolving complex nested SVs through the assembly of pan-genomes across multiple chicken breeds. This work provided valuable insights into the molecular mechanisms underlying avian health, with a particular emphasis on immune-related genes [6]. Furthermore, Wang et al. discovered that transposon factors located in gene bodies or regulatory regions derived from SVs have significant effects on duck domestication and improvement. Notably, a 6945 bp fragment insertion in the *IGF2BP1* gene was associated with body weight, and a 6634 bp insertion in the *MITF* intron may regulate white feather development [7]. Ducks are among the most economically valuable poultry breeds, providing essential products such as eggs, meat, and feathers. To meet the demands of a dynamic market, specific traits of ducks have undergone extensive and intense artificial selection, making it imperative to understand the underlying genetic mechanisms to facilitate breeding efforts. In the previous study, we analyzed the genetic diversity, population structure, and differences in meat breeds of three local breeds in Shandong Province based on single-nucleotide polymorphisms (SNP) loci in order to understand the genetic characteristics of local breed resources. In addition, we explored the potential functional genes responsible for the differentiation of plumage traits between the nascent white feathered population and the original population of Matahu ducks [8]. Compared with SNP loci, which have been studied earlier and applied widely, SV loci are still in their infancy for studying genetic trait differences among breeds. Despite advances in SV research, most studies have focused on major duck breeds. For example, Wang constructed a pan-genome and conducted SV searches in three types of ducks: wild (mallard and mottled duck), local (Jinding duck, Jingjiang mallard, Jinyun mallard, Liancheng white duck, Shanshan mallard, and Youxian mallard), and commercial (Pekin, Cherry Valley, and Grimaud hybrid ducks) [7]. Additionally, Zhang et al. analyzed SVs in Crested ducks, Csp-b SVs in combinations of Crested ducks and Pekin duck genomes, revealing genetic compensatory mechanisms in the anti-tumor and immune systems that are crucial for the survival of crested ducks [9]. Although existing studies have identified SVs associated with specific breed traits, the comprehensive effects of SVs on the genomes and phenotypic traits of local domestic duck breeds remain underexplored due to the diversity of breeds and phenotypes. Thus, further investigation is warranted.

In this study, we employed two software tools, LUMPY and DELLY, to detect and quantify structural variations (SVs) at the whole-genome level in three local domestic duck breeds from Shandong Province: Weishan Partridge Duck (WS), Matahu Duck (MT), and Wendeng Black Duck (WD). We then analyzed the genetic structure of these breeds based on the identified structural variation data. Additionally, we performed a comparative analysis of SVs across the breeds to explore differences in their genomic characteristics. This research enhances our understanding of genome-wide SVs in local domestic duck breeds and provides valuable resources for future genetic characterization and breed selection efforts.

## 2. Materials and Methods

### 2.1. External Characteristics and Basic Production Information of Three Indigenous Domestic Duck Breeds

**Figure 1:**
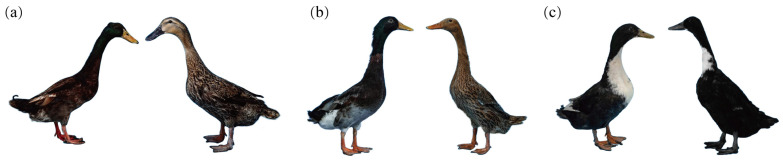
Morphological and external characteristics of local varieties in Shandong Province: (**a**) Weishan partridge duck; (**b**) Matahu duck; (**c**) Wendeng black duck (the left male and the right female).

The external characteristics and basic production information of three indigenous domestic duck breeds from Shandong Province are presented in Figure 1 and Table 1. This information was collected through field visits and research conducted locally.

### 2.2. Data Sources

This study utilized whole-genome resequencing data from 89 ducks representing three distinct breeds (Table 2). Blood samples were obtained through wing vein puncture, with 2 mL of blood collected in EDTA anticoagulation tubes (KWS, Shijiazhuang, China). The samples were transported at 4 °C and subsequently stored at −80 °C. Genomic DNA was extracted from the collected blood samples and sent to the Compass Agritechnology Co., Ltd. (Beijing, China), for sequencing analysis. The qualified libraries were sequenced on DNBSEQ-T7 platform (BGI, Shenzhen, China).

### 2.3. Quality Control and Comparison of Sequencing Data

Clean sequencing data were obtained from the raw bipartite sequencing files through quality control using FASTP software (v0.23.4) [10]. The resulting clean data were aligned to the duck reference genome (GCA_002743455.1) using BWA software (v1.0.6) [11]. Subsequently, the alignment results were processed with SAMBLASTER software (v0.1.26) to remove duplicate reads, add paired-end tags, and establish the maximum number of split reads along with the minimum non-overlapping length [12]. The BAM files were then aligned and sorted, followed by discordant and split-read extraction using SAMTOOLS software (v1.15.1) [13]. For structural variant analysis, the input file consisted of the sorted BAM file generated from the alignment.

### 2.4. Structural Variation Detection

LUMPY and DELLY are widely recognized tools for SV detection. DELLY is particularly sensitive to small SVs, such as insertions and small deletions, whereas LUMPY excels in identifying larger and more complex SVs, including those involving chromosomal rearrangements and structural changes. By integrating the results from both tools, we can overcome the limitations inherent to each method, thereby enhancing the overall accuracy and comprehensiveness of SV detection [14,15]. We analyzed each sample using two identification tools, LUMPY (v0.2.13) and DELLY (v1.0.3), generating a vcf file for each run. It is worth noting that the results from LUMPY were further analyzed for SV genotyping using the SVTyper module. The output from DELLY required conversion from BCF to VCF format. Finally, the SURVIVOR software (v1.0.7) merge tool was employed to integrate and filter the SVs that were identified by both LUMPY and DELLY. The integration process generated the corresponding VCF files using the following parameter settings: 1000 2 1 1 0 30 [16]. Specifically, the parameter settings are as follows: 1000 indicates that the maximum allowable distance for merging SVs is 1000 bp; 2 specifies that only SVs identified by both tools are included; 1 restricts the output to SVs of the same type identified by both tools; the second 1 ensures that only SVs with the same orientation are included; 0 is the default value for an unspecified parameter; and 30 filters out SVs shorter than 30 bp. Additionally, samples from each of the three duck breeds were extracted using PLINK software (v1.9), and custom scripts were used to quantify SVs that were present in all individuals of each breed as well as those that were common in or unique to the three varieties [17]. Genomic annotation files were utilized, and ANNOVAR software “https://annovar.openbioinformatics.org/en/latest/user-guide/download/ (accessed on 22 October 2024)” was employed for genetic annotation [18]. The structural variant files were filtered using VCFTOOLS software (v0.1.16) with the following criteria: --max-missing 0.7 and --maf 0.05 [19].

### 2.5. Analysis of Population Genetic Structure

Principal component analysis (PCA) was conducted using GCTA software (v1.94.0) [20], and the results were visualized based on the first three principal components using the ggplot2 module (v3.5.1) from the R package. For the construction of the neighbor-joining tree, the IBS matrix was first created using PLINK software and visualized with an R package. The resulting NWK file was further embellished using the iTOL website (v7) [21]. Population structure analysis was performed with ADMIXTURE software (v1.3.0) [22], and the grep command was utilized to extract the cross-validation error rate from the log file to determine the optimal subpopulation inference, which was subsequently visualized using the pophelper module (v2.3.1) of the R package [23].

### 2.6. Selection Signal Analysis

VCFTOOLS software was used to calculate the genetic differentiation index (Fst) for any two of the three breeds. Parameters were set for a 50 Kb window with a 20 Kb step size. First, we calculated the differentiation indices among the three varieties based on the “WEIGHTED_FST” column in the result file. Next, the Fst values were sorted in descending order, and the top 5% of SVs were identified as candidate regions exhibiting a higher degree of genomic differentiation between the populations. At the same time, we used the g:Profiler online website “https://biit.cs.ut.ee/gprofiler/convert (accessed on 24 October 2024)” for gene annotation of the obtained candidate SVs [24].

### 2.7. Functional Gene Enrichment Analysis

Gene ontology (GO) enrichment analyses were conducted using the ClusterProfiler package (v4.14.1) in R [25], while Kyoto Encyclopedia of Genes and Genomes (KEGG) enrichment analyses were performed using the KOBAS web service “http://bioinfo.org/kobas/genelist/ (accessed on 28 October 2024)” [26]. Statistical assessments employed the hypergeometric test/Fisher’s exact test, with false discovery rate (FDR) corrections based on the method proposed by Benjamini and Hochberg [27]. Meanwhile, we utilized the STRING database (https://cn.string-db.org/; accessed on 30 October 2024) to conduct a protein–protein interaction network analysis. This approach enabled us to identify key protein molecules involved in stress resistance, growth, and development, as well as to explore their associated functional genes [28].

## 3. Results

### 3.1. Genome-Wide Structural Variation Detection and Distribution Statistics

**Figure 2:**
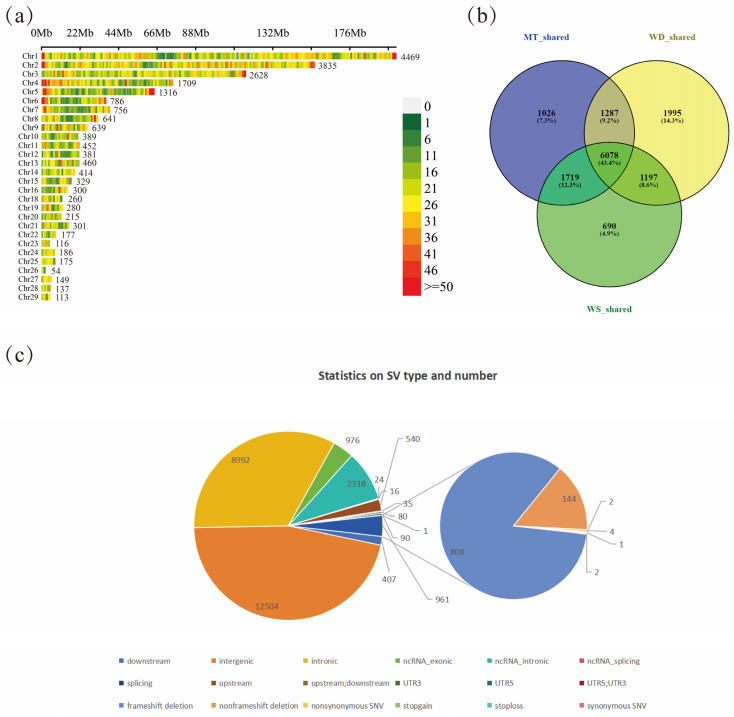
Identification of SVs in local domestic duck breeds in Shandong: (**a**) number distribution of SVs on chromosomes; (**b**) distribution of SV numbers in different groups; (**c**) position distribution of SVs on the genome.

After the raw data were quality tested and filtered, the three populations obtained an average of 45 Gb of clean base per sample, with 99% and 96% Q20 and Q30, respectively, and an average GC content of 41%. To construct a comprehensive SV dataset, we analyzed sequencing data from 89 local domestic duck individuals using two software programs, LUMPY and DELLY, for SVs identification. A total of 21,673 SVs were identified in this analysis, with an average of 240 SVs per individual. Compared to other studies, the number of SVs identified in this study is relatively modest. For instance, Wang et al. reported the identification of 101,041 SVs across 131 duck genomes, yielding an average of 770 SVs per individual [7]. This inconsistency can be attributed, in part, to differences in the number of varieties and individuals included in the analysis. Additionally, factors such as the construction of the pan-genome and the use of various SV detection tools also influence the total number of SVs identified. Chromosome 1 exhibited the highest number of SVs (4469), while chromosome 26 had the lowest (54), indicating an uneven distribution of SVs across chromosomes (Figure 2a). This variation correlates with chromosome length and mirrors the distribution patterns observed for SNPs and runs of homozygosity (ROH). We found that the higher SV densities were mainly located at the ends of the long chromosomes, with particularly notable expressions on chr1, 2, and 5. In general, chromosome length appeared to be positively correlated with the distribution and frequency of SVs. However, this relationship is influenced by multiple factors, including but not limited to the physical length of the chromosome, the distribution of functional regions, recombination rates, and the methodologies employed for SV detection. Additionally, we analyzed the total number of SVs identified across all individuals from the three varieties—MT, WS, and WD—finding 10,110, 9684, and 10,557 SVs, respectively. The number of variety-specific SVs was 1026 for MT, 690 for WS, and 6078 for WD, while 1995 SVs were shared across all three varieties (Figure 2b). Regarding SV types, the majority (46%, or 9460 SVs) were located in intergenic regions. Intronic regions contained the second highest proportion of SVs at 33%. Within exonic cregions, frameshift deletions were the most prevalent, accounting for 3% of the total SVs, followed by non-frameshift deletions at 1% (Figure 2; Tables S1 and S2).

### 3.2. Analysis of Population Structure

**Figure 3:**
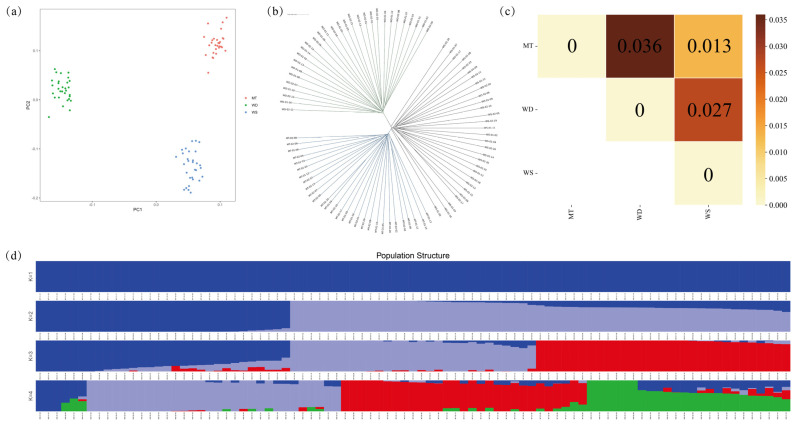
Population structure analysis of three local domestic duck breeds: (**a**) principal component analysis; (**b**) neighbor-joining tree; (**c**) population differentiation indices; (**d**) population structure analysis. K: number of subgroups of the population.

Analysis of the principal component results reveals that the individuals from the three groups are distinctly separated, with minimal overlap, indicating that the groups are relatively independent. This separation suggests that the samples are appropriately assigned to their respective groups (Figure 3a). The eigenvalues for the first three principal components are 3.60146, 1.47835, and 0.816043, respectively, accounting for 61.02%, 25.42%, and 13.56% of the total variance. Together, the first two principal components explain over 80% of the variance in the data, demonstrating that principal components 1 and 2 effectively capture the underlying structure of the dataset. The WS and MT ducks exhibited greater dispersion compared to the WD duck. Similarly, neighbor-joining tree analyses supported these findings, showing each group as distinct with no mixed individuals between groups (Figure 3b). Notably, four individuals from the WS population did not cluster with the majority of the other individuals from the same group but were instead distributed around the cluster. This observation suggests potential genetic heterogeneity within the WS population, or the possibility that these individuals may have originated from a subpopulation. The close proximity of the WS and MT groups on the dendrogram further implies a close genetic relationship between these two populations. Genetic differentiation between the three populations was assessed using inter-population differentiation indices. The genetic distances were 0.036 between MT and WD, 0.013 between MT and WS, and 0.027 between WD and WS. These values indicate low to moderate levels of genetic differentiation, suggesting that the populations are genetically similar. Such differentiation may be attributable to factors such as geographic isolation, environmental influences, or subtle selective pressures (Figure 3c).

Population structure analysis identified the lowest cross-validation (CV) value at K (Number of subgroups of the population) = 2, suggesting that the three breeds are best represented by two ancestral components. At K = 2, MT and WD appear to share a common ancestral lineage, with WD showing relative isolation (Figure 3d). At K = 3, all 89 ducks were accurately assigned to one of three distinct taxa. However, approximately half of the individuals exhibited a slight degree of admixture, with evidence of exogenous ancestry, such as MT ancestry within the WS population. This admixture suggests that gene flow may have occurred between populations, either recently or in the historical past. However, at K = 4, evidence of admixture was observed in the WS population, indicating the potential introduction of foreign genetic material.

### 3.3. Analysis of Selection Signals Among Local Domestic Duck Breeds

**Figure 4:**
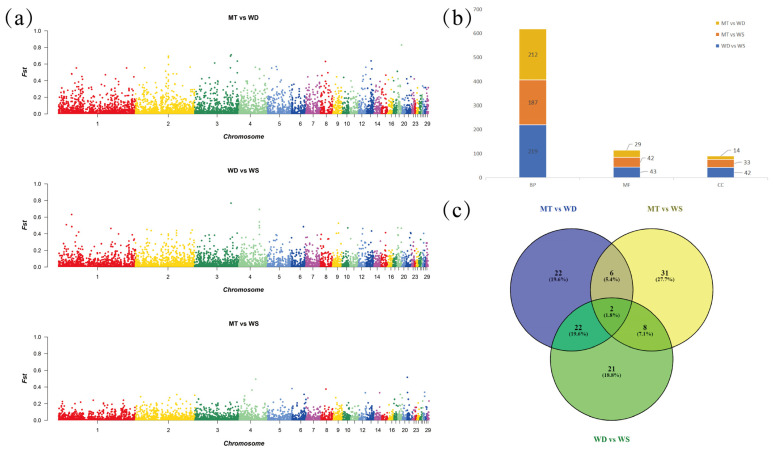
Differential subject to selection analysis among different subgroups of Shandong local domestic duck breeds: (**a**) Manhattan plot on the analysis of Fst method; (**b**) distribution of the number of GO terms in different subgroups; (**c**) shared number of genes related to the development of the nervous system in different subgroups. BP: biological process; CC: cellular component; molecular function.

In this study, we first filtered the VCF file with parameters set to MAF of 0.05 and a ---max-missing rate of 0.7. As the count went on, 21,673 SVs ended up with 9125 SVs being retained. We then applied the Fst method to detect SV differences among the three duck breeds (Figure 4a). The SVs with the top 5% Fst values were selected as candidate regions for analysis. Our findings showed that 883 selected SV loci between MT and WS ducks, 1004 selected SV loci between MT and WD ducks, and 891 selected SV loci between WS and WD ducks. To investigate the impact of these SVs on gene function, we successfully annotated 473, 601, and 493 genes using the g:Convert tool from the g:Profiler website, for each comparison, respectively. These genes were then subjected to GO and KEGG enrichment analyses (Tables S3 and S4). Significantly enriched pathways were identified using a threshold of *p* < 0.05. A total of 304, 262, and 255 significantly enriched GO terms were identified in the WD vs. WS, MT vs. WS, and MT vs. WD comparisons, respectively (Figure 4b). In contrast, fewer significant KEGG pathways were identified, with only nine pathways detected across all three subgroups.

#### 3.3.1. Analysis of Enriched GO Terms and KEGG Pathways

**Figure 5:**
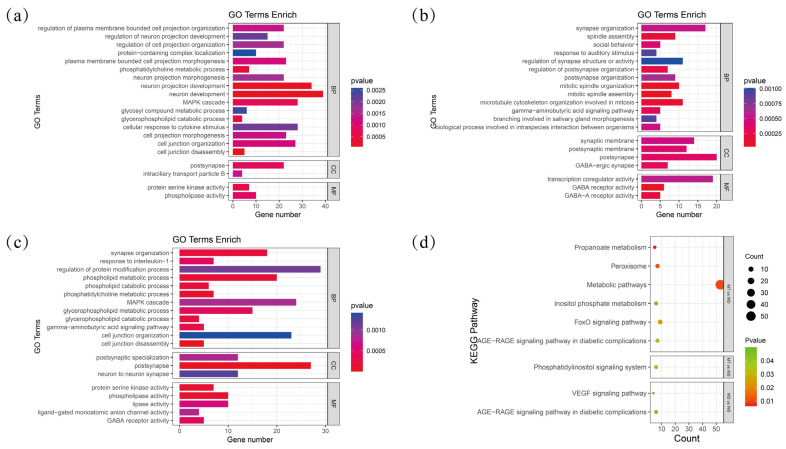
Functional enrichment analysis of genes in different subgroups: (**a**) GO enrichment analysis in the subgroups of MT and WD; (**b**) GO enrichment analysis in the subgroups of MT and WS; (**c**) GO enrichment analysis in the subgroups of WD and WS; (**d**) KEGG enrichment analysis in the subgroups of MT and WD, MT and WS, and WD and WS.

We performed a statistical analysis of the top 20 GO terms across three subgroups (Figure 5). In the MT and WD subgroups, numerous terms were related to neurodevelopment, including neuron development (GO:0048666), neuron projection development (GO:0031175), neuron projection morphogenesis (GO:0048812), and regulation of neuron projection development (GO:0010975). We have identified a substantial number of pathways and associated genes related to neurodevelopment (Figure 4c). However, these findings are based solely on genomic data, highlighting genetic differences between the populations. To date, there are no studies addressing neurodevelopmental or behavioral differences among local domestic duck breeds, and the underlying regulatory mechanisms remain to be explored.

In our survey (Table 1), we observed significant differences in egg production, egg quality, and hatchability among the three local breeds. The WD breed exhibits earlier onset of laying and higher annual egg production compared to the WS and MT breeds. Notably, WS ducks demonstrate greater egg weight and hatchability, which may be attributable to the enhanced nutritional composition of the eggshell. In the MT and WS subgroups, terms associated with mitosis and spindle assembly were heavily enriched, such as mitotic spindle assembly (GO:0090307), mitotic spindle organization (GO:0007052), microtubule cytoskeleton organization involved in mitosis (GO:1902850), and spindle assembly (GO:0051225). Additionally, terms related to synapse structure or activity were significantly enriched, including transcription coregulator activity (GO:0003712), synaptic membrane (GO:0097060), postsynaptic membrane (GO:0045211), and regulation of synapse structure or activity (GO:0050803).

Based on the findings, in terms of growth rate, the overall body weight of the WD ducks at the eighth week was greater than that of the MT and WS ducks. However, the slaughter yield during this period was comparable across all groups. These results suggest that the WD ducks exhibit a relative advantage in terms of growth performance while demonstrating similar slaughter efficiency to the other groups. In the WD and WS subgroups, terms related to energy metabolism were significantly associated, such as phospholipid metabolic process (GO:0006644), glycerophospholipid catabolic process (GO:0046475), phospholipid catabolic process (GO:0009395), and glycerophospholipid metabolic process (GO:0006650). Notably, the term neuron development (GO:0048666) was enriched with the highest number of genes across all three groups, with 39, 30, and 29 genes in each group, respectively (Figure 3c).

In the KEGG pathway enrichment analysis, the MT and WD subgroups exhibited the highest number of enriched pathways, primarily related to energy metabolism. Key pathways included propanoate metabolism (apla00640), metabolic pathways (apla01100), and peroxisome (apla04146), with metabolic pathways showing the highest gene enrichment. In the MT and WS subgroups, the phosphatidylinositol signaling system (apla04070) was associated with signal transduction. In the WD and WS subgroups, the AGE-RAGE signaling pathway in diabetic complications (apla04933) and VEGF signaling pathway (apla04370) were linked to energy metabolism and angiogenesis, respectively.

#### 3.3.2. Protein–Protein Interaction (PPI) Network Analysis

**Figure 6:**
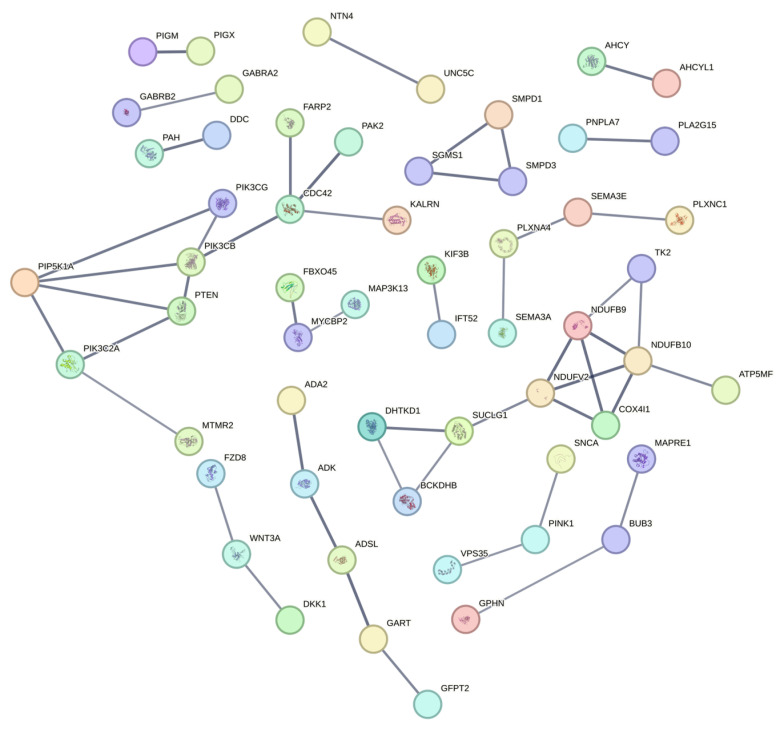
Protein interaction network (PPI) of genes related to nervous system development, mitosis and spindle assembly, and energy metabolism.

The interplay between nervous system development and tissue metabolic activity represents a complex and interesting biological process. In this study, we sought to identify common protein molecules and their associated functional genes involved in neuromodulation, energy metabolism, and reproductive development. This was achieved through a network analysis of protein–protein interactions. Our findings aim to enhance the understanding of the regulatory mechanisms underlying economically significant traits in local domestic duck breeds, with implications for their selection and breeding. We utilized the STRING database to perform a PPI network analysis on 207 genes associated with key biological processes in the three duck species (Figure 6). These processes included neural development (GO term), mitotic spindle assembly activities (in the MT and WS subgroups), and energy metabolism (in the WD and WS subgroups). After removing isolated nodes and setting a high confidence threshold of 0.700, we identified two significant protein interaction networks. The first network included *CDC42*, *PIK3CB*, *PIP5K1A*, *PIK3CG*, *PTEN*, and *PIK3C2A*. The second network comprised *NDUFB9*, *NDUFB10*, *NDUFV2*, *COX4I1*, and *TK2*. Literature review indicates that *CDC42* may serve a critical bridging role in both neural development and energy metabolism. This suggests that *CDC42* could be a key regulatory protein influencing multiple essential biological pathways in these duck populations.

## 4. Discussion

In this study, we employed two software programs, LUMPY and DELLY, to detect SVs with the aim of enhancing detection accuracy and minimizing false positives. Our analysis identified a total of 21,673 SVs across three local domestic duck breeds. The comparison of SVs among these breeds underscored the complexity of SVs in the genomes of local domestic duck breeds.

Genomic variants are categorized into three primary types: SNPs, insertions/deletions (Indels), and SVs [29]. Numerous studies have demonstrated that SVs are prevalent in genomes and exert significant phenotypic effects, often explaining population diversity more effectively than SNPs [30]. However, it is crucial to note that most SVs are not directly associated with trait manifestation but rather with environmental responses or other phenotypic polymorphisms [31]. In this study, we analyzed the genetic clustering of three local domestic duck breeds in Shandong. Both PCA and neighbor-joining tree results indicated that the three groups are relatively independent, forming distinct taxa. Population structure analysis suggested that MT and WS ducks might share a common ancestor, while WD ducks are genetically distinct, likely due to their geographical isolation limiting gene flow.

Poultry are highly sensitive to environmental changes and external stimuli, which can lead to reproductive issues, growth disruptions, decreased egg-laying performance, and increased susceptibility to diseases, ultimately reducing production efficiency. During domestication, individuals with docile temperaments and lower stress responses are typically selected for breeding, leading to the gradual selection and fixation of genes associated with nervous system development [32]. In our study, we identified 112 genes related to neurodevelopment by screening the three subgroups for GO terms. Among these, 38 genes were detected in at least two subgroups, indicating that different breeds may share key neurodevelopment-related genes, while also exhibiting breed-specific genetic variations. These variations could contribute to breed-specific neurodevelopmental traits, making neurodevelopmental genes potential markers for distinguishing phenotypic traits between breeds [33].

Genes such as plexin A4 (*PLXNA4*), neuropilin 2 (*NRP2*), and members of the nerve growth factor family, including semaphorin 3A (*SEMA3A*) and semaphorin 3C (*SEMA3C*), may play roles in neural development and synaptic plasticity [34,35,36,37]. Additionally, calcium/calmodulin dependent protein kinase ID (*CAMK1D*), glutaredoxin and cysteine rich domain containing 1 (*GRXCR1*), and FBXO protein 45 (*FBXO45*) are implicated in cellular stress responses, signaling, cell cycle regulation, and stress responses [38,39,40]. Gamma-aminobutyric acid type a receptor subunit alpha2 (*GABRA2*) and gamma-aminobutyric acid type a receptor subunit beta2 (*GABRB2*)—which encode GABA receptor subunits—along with adhesion G protein-coupled receptor B1 (*ADGRB1*) are involved in stress response and emotion regulation [41,42]. Ankyrin repeat domain 1 (*ANKRD1*) is associated with stress and cardiovascular health [43].

GO terms related to spindle assembly were enriched in the MT and WS subgroups. Proper spindle function is essential for egg-laying, as it ensures correct oocyte segregation during mitosis, affecting egg quality and quantity. Genes such as phosphatase and tensin homolog (*PTEN*), *MYB* proto-oncogene like 2 (*MYBL2*), and cell division cycle 42 (*CDC42*) are involved in cell signaling and cell cycle regulation, impacting germ cell development and reproduction [44,45,46]. Kinesin family member 3B (*KIF3B*) and targeting protein for xklp2 (*TPX2*) are crucial for intracellular transport, microtubule organization, and spindle formation, affecting germ cell health and division [47,48]. *TPX2*, in particular, shows high expression during porcine oocyte stages, with knockdown leading to meiotic cycle progression issues and spindle abnormalities [49].

Energy-metabolism-related GO and KEGG pathways were significantly enriched in the WD and WS subgroups. We annotated 86 genes across 14 GO terms and 3 KEGG pathways related to energy metabolism. Genes such as adenosine kinase (*ADK*), pantothenate kinase 1 (*PANK1*), *PTEN*, PTEN induced kinase 1 (*PINK1*) and cytochrome c oxidase subunit 4I1 (*COX4I1*) regulate ovarian function, embryonic development, cellular respiration, cell proliferation, and apoptosis, all of which are critical for egg-laying performance [50,51,52,53,54].

Differences in the expression of key neurodevelopmental genes among breeds may influence specific traits or phenotypes. We highlight the potential impact of the nervous system on energy metabolism and egg-laying activity. Stress-induced sympathetic nervous system activation increases adrenaline and cortisol secretion, redirecting energy usage towards stress responses rather than reproduction, thus affecting egg-laying performance [55]. Additionally, the nervous system regulates energy intake and expenditure through the hypothalamus, influencing reproductive hormone secretion and energy metabolism via hormones like insulin, leptin, and adrenaline [56]. Protein kinase cGMP-dependent 1 (*PRKG1*), *GABRA2*, ETS variant transcription factor 1 (*ETV1*), and myelin transcription factor 1 like (*MYT1L*) are associated with neurodevelopment and endocrine regulation [57,58,59]. *PRKG1*, involved in lipolysis and muscle fatty acid composition, is significant in animals like pigs, sheep, and cattle [60,61,62]. Follicle-stimulating hormone receptor (*FSHR*), which binds follicle-stimulating hormone in the ovary, affects follicle development and hormone secretion [63]. *CDC42*, a small GTPase, plays a pivotal role in cell signaling, migration, and morphology and is crucial for nervous system development, female reproduction, and energy metabolism [64,65,66].

Advances in genome-sequencing technologies and the improved detection of structural variations in ducks have significantly enhanced our understanding of the genetic underpinnings of key resource characteristics and production traits. However, elucidating the genetic basis of the variation in economically important traits among different breeds remains a complex challenge, particularly when it comes to subtle phenotypic differences within a single breed. The molecular mechanisms driving these variations require further investigation and clarification. In this study, we conducted a comparative analysis of structural variations across three duck breeds at the genomic level, identifying potential candidate genes associated with neural development, reproductive performance, and energy metabolism. This investigation represents an initial step in uncovering the genetic factors contributing to these traits. However, we recommend that future studies incorporate population-wide assessments of specific SVs. Additionally, we suggest that genome-wide association studies (GWAS) and cellular functional validation be carried out in future research to further clarify the mechanisms of action. Such studies will be crucial for refining breeding strategies aimed at improving the productivity and health of local domestic duck breeds.

## 5. Conclusions

In this study, we identified genomic SVs in local domestic duck breeds from Shandong using whole genome sequencing data. Our analysis of these SVs revealed the genetic relationships among three duck populations, highlighting a close relationship between MT and WS. Through selection signal analysis, we identified structural variants associated with domestication traits, with genes involved in nervous system development, spindle assembly, and energy metabolism processes. These findings contribute to a deeper understanding of the genetic characteristics of local domestic duck breeds, particularly the SVs linked to economically important traits. Future studies should focus on the functional validation of the identified structural variants at both the population and molecular levels. This will enable a more comprehensive understanding of the specific roles these SVs play in the regulation of traits such as reproduction, growth, and metabolism. Ultimately, these efforts will provide valuable genetic resources to improve the selection, breeding, and conservation of local domestic duck breeds, enhancing their economic potential and sustainability.

## Figures and Tables

**Table 1:** Basic information related to the production of three local domestic duck breeds in Shandong Province.

Duck Breeds ID	Weight at 1 Day Old (g)	Weight at 8 Weeks Old (g)	8-Week-Old Slaughter Rate (%)	Age at Start of Production (Days)	Annual Egg Production	Average Egg Weight (g)	Incubation Rate (%)
WS	47.8/47.3	1419.7/1329.3	88.1/87.3	140	180–200	80	93
MT	50.7/48.4	1479.0/1421.7	89.7/87.8	140	180–200	70	85
WD	52.1/50.9	1724.8/1569.2	88.7/87.3	130	210–240	75	90

Note: The weight at 1 day old, weight at 8 weeks old, and 8-week-old slaughter rate are presented as male/female controls (male/female). WS: Weishan partridge duck; MT: Matahu duck; WD: Wendeng black duck (the same as below).

**Table 2:** Test sample collection information.

Duck Breeds ID	Number	Station
WS	30	Jining Weishan Xinhe Laying Duck Breeding Co.
MT	29	
WD	30	Weihai Qinghe Wendeng Black Duck Original Breeding Farm

## Data Availability

The datasets analyzed in this study are available from the corresponding author upon reasonable request.

## Supplementary Materials

The following supporting information can be downloaded at: https://www.mdpi.com/article/10.3390/ani14243657/s1, Table S1: Location information for structural variation detection results; Table S2: Exon region information; Table S3: GO enrichment analysis on the results of selection signal analysis among different varieties; Table S4: KEGG enrichment analysis on the results of selection signal analysis among different species.

## References

[1] Zhang Z., Van Treuren R., Yang T., Hu Y., Zhou W., Liu H., Wei T. (2023). A Comprehensive Lettuce Variation Map Reveals the Impact of Structural Variations in Agronomic Traits. BMC Genom..

[2] Huang Y., Wang H., Xu S., Liu J., Zeng Q., Hu J., Bao Z. (2024). Identification of Structural Variation Related to Spawn Capability of Penaeus Vannamei. BMC Genom..

[3] Ben-Jemaa S., Boussaha M., Mandonnet N., Bardou P., Naves M. (2024). Uncovering Structural Variants in Creole Cattle from Guadeloupe and Their Impact on Environmental Adaptation through Whole Genome Sequencing. PLoS ONE.

[4] Liu X., Liu W., Lenstra J.A., Zheng Z., Wu X., Yang J., Li B., Yang Y., Qiu Q., Liu H. (2023). Evolutionary Origin of Genomic Structural Variations in Domestic Yaks. Nat. Commun..

[5] Kwon D., Park N., Wy S., Lee D., Park W., Chai H.-H., Cho I.-C., Lee J., Kwon K., Kim H. (2024). Identification and Characterization of Structural Variants Related to Meat Quality in Pigs Using Chromosome-Level Genome Assemblies. BMC Genom..

[6] Rice E.S., Alberdi A., Alfieri J., Athrey G., Balacco J.R., Bardou P., Blackmon H., Charles M., Cheng H.H., Fedrigo O. (2023). A Pangenome Graph Reference of 30 Chicken Genomes Allows Genotyping of Large and Complex Structural Variants. BMC Biol..

[7] Wang K., Hua G., Li J., Yang Y., Zhang C., Yang L., Hu X., Scheben A., Wu Y., Gong P. (2024). Duck Pan-genome Reveals Two Transposon Insertions Caused Bodyweight Enlarging and White Plumage Phenotype Formation during Evolution. iMeta.

[8] Ren P., Yang L., Khan M.Z., Jing Y., Zhang M., Qi C., Zhang X., Liu X., Liu Z., Zhang S. (2024). Joint Genomic and Transcriptomic Analysis Reveals Candidate Genes Associated with Plumage Color Traits in Matahu Ducks. Animals.

[9] Chang G., Yuan X., Guo Q., Bai H., Cao X., Liu M., Wang Z., Li B., Wang S., Jiang Y. (2023). The First Crested Duck Genome Reveals Clues to Genetic Compensation and Crest Cushion Formation. Genom. Proteom. Bioinform..

[10] Chen S., Zhou Y., Chen Y., Gu J. (2018). Fastp: An Ultra-Fast All-in-One FASTQ Preprocessor. Bioinformatics.

[11] Li H., Durbin R. (2009). Fast and Accurate Short Read Alignment with Burrows–Wheeler Transform. Bioinformatics.

[12] Faust G.G., Hall I.M. (2014). SAMBLASTER: Fast Duplicate Marking and Structural Variant Read Extraction. Bioinformatics.

[13] Li H., Handsaker B., Wysoker A., Fennell T., Ruan J., Homer N., Marth G., Abecasis G., Durbin R., 1000 Genome Project Data Processing Subgroup (2009). The Sequence Alignment/Map Format and SAMtools. Bioinformatics.

[14] Layer R.M., Chiang C., Quinlan A.R., Hall I.M. (2014). LUMPY: A Probabilistic Framework for Structural Variant Discovery. Genome Biol..

[15] Rausch T., Zichner T., Schlattl A., Stütz A.M., Benes V., Korbel J.O. (2012). DELLY: Structural Variant Discovery by Integrated Paired-End and Split-Read Analysis. Bioinformatics.

[16] Jeffares D.C., Jolly C., Hoti M., Speed D., Shaw L., Rallis C., Balloux F., Dessimoz C., Bähler J., Sedlazeck F.J. (2017). Transient Structural Variations Have Strong Effects on Quantitative Traits and Reproductive Isolation in Fission Yeast. Nat. Commun..

[17] Purcell S., Neale B., Todd-Brown K., Thomas L., Ferreira M.A.R., Bender D., Maller J., Sklar P., de Bakker P.I.W., Daly M.J. (2007). PLINK: A Tool Set for Whole-Genome Association and Population-Based Linkage Analyses. Am. J. Hum. Genet..

[18] Wang K., Li M., Hakonarson H. (2010). ANNOVAR: Functional Annotation of Genetic Variants from High-Throughput Sequencing Data. Nucleic Acids Res..

[19] Danecek P., Auton A., Abecasis G., Albers C.A., Banks E., DePristo M.A., Handsaker R.E., Lunter G., Marth G.T., Sherry S.T. (2011). The Variant Call Format and VCFtools. Bioinformatics.

[20] Yang J., Lee S.H., Goddard M.E., Visscher P.M. (2011). GCTA: A Tool for Genome-Wide Complex Trait Analysis. Am. J. Hum. Genet..

[21] Letunic I., Bork P. (2024). Interactive Tree of Life (iTOL) v6: Recent Updates to the Phylogenetic Tree Display and Annotation Tool. Nucleic Acids Res..

[22] Alexander D.H., Novembre J., Lange K. (2009). Fast Model-Based Estimation of Ancestry in Unrelated Individuals. Genome Res..

[23] Francis R.M. (2017). pophelper: An R Package and Web App to Analyse and Visualize Population Structure. Mol. Ecol. Resour..

[24] Kolberg L., Raudvere U., Kuzmin I., Adler P., Vilo J., Peterson H. (2023). G:Profiler—Interoperable Web Service for Functional Enrichment Analysis and Gene Identifier Mapping (2023 Update). Nucleic Acids Res..

[25] Wu T., Hu E., Xu S., Chen M., Guo P., Dai Z., Feng T., Zhou L., Tang W., Zhan L. (2021). clusterProfiler 4.0: A Universal Enrichment Tool for Interpreting Omics Data. Innovation.

[26] Bu D., Luo H., Huo P., Wang Z., Zhang S., He Z., Wu Y., Zhao L., Liu J., Guo J. (2021). KOBAS-i: Intelligent Prioritization and Exploratory Visualization of Biological Functions for Gene Enrichment Analysis. Nucleic Acids Res..

[27] Benjamini Y. (2010). Discovering the False Discovery Rate. J. R. Stat. Soc..

[28] Szklarczyk D., Kirsch R., Koutrouli M., Nastou K., Mehryary F., Hachilif R., Gable A.L., Fang T., Doncheva N.T., Pyysalo S. (2023). The STRING Database in 2023: Protein–Protein Association Networks and Functional Enrichment Analyses for Any Sequenced Genome of Interest. Nucleic Acids Res..

[29] Wu Z., Jiang Z., Li T., Xie C., Zhao L., Yang J., Ouyang S., Liu Y., Li T., Xie Z. (2021). Structural Variants in the Chinese Population and Their Impact on Phenotypes, Diseases and Population Adaptation. Nat. Commun..

[30] Lee Y.-L., Bosse M., Takeda H., Moreira G.C.M., Karim L., Druet T., Oget-Ebrad C., Coppieters W., Veerkamp R.F., Groenen M.A.M. (2023). High-Resolution Structural Variants Catalogue in a Large-Scale Whole Genome Sequenced Bovine Family Cohort Data. BMC Genom..

[31] Mahmoud M., Gobet N., Cruz-Dávalos D.I., Mounier N., Dessimoz C., Sedlazeck F.J. (2019). Structural Variant Calling: The Long and the Short of It. Genome Biol..

[32] Zhang X., Wang K., Wang L., Yang Y., Ni Z., Xie X., Shao X., Han J., Wan D., Qiu Q. (2016). Genome-Wide Patterns of Copy Number Variation in the Chinese Yak Genome. BMC Genom..

[33] Kennedy A., Weissbourd B. (2024). Dynamics of Neural Activity in Early Nervous System Evolution. Curr. Opin. Behav. Sci..

[34] Danelon V., Goldner R., Martinez E., Gokhman I., Wang K., Yaron A., Tran T.S. (2020). Modular and Distinct Plexin-A4/FARP2/Rac1 Signaling Controls Dendrite Morphogenesis. J. Neurosci..

[35] Koropouli E., Wang Q., Mejías R., Hand R., Wang T., Ginty D.D., Kolodkin A.L. (2023). Palmitoylation Regulates Neuropilin-2 Localization and Function in Cortical Neurons and Conveys Specificity to Semaphorin Signaling via Palmitoyl Acyltransferases. eLife.

[36] Carulli D., De Winter F., Verhaagen J. (2021). Semaphorins in Adult Nervous System Plasticity and Disease. Front. Synaptic Neurosci..

[37] Vieira J.R., Shah B., Dupraz S., Paredes I., Himmels P., Schermann G., Adler H., Motta A., Gärtner L., Navarro-Aragall A. (2022). Endothelial PlexinD1 Signaling Instructs Spinal Cord Vascularization and Motor Neuron Development. Neuron.

[38] Grant P., Kumar J., Kar S., Overduin M. (2021). Effects of Specific Inhibitors for CaMK1D on a Primary Neuron Model for Alzheimer’s Disease. Molecules.

[39] Steffens D.C., Garrett M.E., Soldano K.L., McQuoid D.R., Ashley-Koch A.E., Potter G.G. (2020). Genome-Wide Screen to Identify Genetic Loci Associated with Cognitive Decline in Late-Life Depression. Int. Psychogeriatr..

[40] Na Y., Calvo-Jiménez E., Kon E., Cao H., Jossin Y., Cooper J.A. (2020). Fbxo45 Binds SPRY Motifs in the Extracellular Domain of N-Cadherin and Regulates Neuron Migration during Brain Development. Mol. Cell. Biol..

[41] Ionescu-Tucker A., Butler C.W., Berchtold N.C., Matheos D.P., Wood M.A., Cotman C.W. (2021). Exercise Reduces H3K9me3 and Regulates Brain Derived Neurotrophic Factor and GABRA2 in an Age Dependent Manner. Front. Aging Neurosci..

[42] Hamanaka K., Miyake N., Mizuguchi T., Miyatake S., Uchiyama Y., Tsuchida N., Sekiguchi F., Mitsuhashi S., Tsurusaki Y., Nakashima M. (2022). Large-Scale Discovery of Novel Neurodevelopmental Disorder-Related Genes through a Unified Analysis of Single-Nucleotide and Copy Number Variants. Genome Med..

[43] Xu X., Wang X., Li Y., Chen R., Wen H., Wang Y., Ma G. (2024). Research Progress of Ankyrin Repeat Domain 1 Protein: An Updated Review. Cell. Mol. Biol. Lett..

[44] Su C., Zhang R., Zhang X., Lv M., Liu X., Ao K., Hao J., Mu Y. (2023). Dingkun Pill Modulate Ovarian Function in Chemotherapy-Induced Premature Ovarian Insufficiency Mice by Regulating PTEN/PI3K/AKT/FOXO3a Signaling Pathway. J. Ethnopharmacol..

[45] Zhang J., Liu W., Li G., Xu C., Nie X., Qin D., Wang Q., Lu X., Liu J., Li L. (2022). BCAS2 Is Involved in Alternative Splicing and Mouse Oocyte Development. FASEB J..

[46] Xu W., Yuan Y., Tian Y., Cheng C., Chen Y., Zeng L., Yuan Y., Li D., Zheng L., Luo T. (2023). Oral Exposure to Polystyrene Nanoplastics Reduced Male Fertility and Even Caused Male Infertility by Inducing Testicular and Sperm Toxicities in Mice. J. Hazard. Mater..

[47] Heydari R., Seresht-Ahmadi M., Mirshahvaladi S., Sabbaghian M., Mohseni-Meybodi A. (2022). KIF3B Gene Silent Variant Leading to Sperm Morphology and Motility Defects and Male Infertility. Biol. Reprod..

[48] Naso F.D., Sterbini V., Crecca E., Asteriti I.A., Russo A.D., Giubettini M., Cundari E., Lindon C., Rosa A., Guarguaglini G. (2020). Excess TPX2 Interferes with Microtubule Disassembly and Nuclei Reformation at Mitotic Exit. Cells.

[49] He Y., Peng L., Li J., Li Q., Chu Y., Lin Q., Rui R., Ju S. (2022). TPX2 Deficiency Leads to Spindle Abnormity and Meiotic Impairment in Porcine Oocytes. Theriogenology.

[50] Li H., Zheng J., Xu Q., Yang Y., Zhou J., Guo X., Cai Y., Cai J.J., Xie L., Awika J. (2023). Hepatocyte Adenosine Kinase Promotes Excessive Fat Deposition and Liver Inflammation. Gastroenterology.

[51] Li S.-S., Liu Q.-J., Bao J.-X., Lu M., Deng B.-Q., Li W.-W., Cao C.-C. (2024). Counteracting TGM2 by a Fibroin Peptide Ameliorated Adriamycin-Induced Nephropathy via Regulation of Lipid Metabolism through PANX1-PPAR α/PANK1 Pathway. Transl. Res..

[52] Endicott S.J., Miller R.A. (2024). PTEN Activates Chaperone-Mediated Autophagy to Regulate Metabolism. Autophagy.

[53] Li Y., Chen H., Xie X., Yang B., Wang X., Zhang J., Qiao T., Guan J., Qiu Y., Huang Y.-X. (2023). PINK1-Mediated Mitophagy Promotes Oxidative Phosphorylation and Redox Homeostasis to Induce Drug-Tolerant Persister Cancer Cells. Cancer Res..

[54] Čunátová K., Reguera D.P., Vrbacký M., Fernández-Vizarra E., Ding S., Fearnley I.M., Zeviani M., Houštěk J., Mráček T., Pecina P. (2021). Loss of COX4I1 Leads to Combined Respiratory Chain Deficiency and Impaired Mitochondrial Protein Synthesis. Cells.

[55] Ghasemi M., Mehranfard N. (2024). Neuroprotective Actions of Norepinephrine in Neurological Diseases. Pflügers Arch.-Eur. J. Physiol..

[56] Sandoval D.A., Gong B., Davis S.N. (2007). Antecedent Short-Term Central Nervous System Administration of Estrogen and Progesterone Alters Counterregulatory Responses to Hypoglycemia in Conscious Male Rats. Am. J. Physiol.-Endocrinol. Metab..

[57] Tahir M.S., Porto-Neto L.R., Gondro C., Shittu O.B., Wockner K., Tan A.W.L., Smith H.R., Gouveia G.C., Kour J., Fortes M.R.S. (2021). Meta-Analysis of Heifer Traits Identified Reproductive Pathways in Bos Indicus Cattle. Genes.

[58] Ben-Jemaa S., Senczuk G., Ciani E., Ciampolini R., Catillo G., Boussaha M., Pilla F., Portolano B., Mastrangelo S. (2021). Genome-Wide Analysis Reveals Selection Signatures Involved in Meat Traits and Local Adaptation in Semi-Feral Maremmana Cattle. Front. Genet..

[59] Wöhr M., Fong W.M., Janas J.A., Mall M., Thome C., Vangipuram M., Meng L., Südhof T.C., Wernig M. (2022). Myt1l Haploinsufficiency Leads to Obesity and Multifaceted Behavioral Alterations in Mice. Mol. Autism.

[60] Zhang W., Li X., Jiang Y., Zhou M., Liu L., Su S., Xu C., Li X., Wang C. (2022). Genetic Architecture and Selection of Anhui Autochthonous Pig Population Revealed by Whole Genome Resequencing. Front. Genet..

[61] Ren Y., Chen X., Zheng X., Wang F., Sun R., Wei L., Zhang Y., Liu H., Lin Y., Hong L. (2023). Diverse WGBS Profiles of Longissimus Dorsi Muscle in Hainan Black Goats and Hybrid Goats. BMC Genom Data.

[62] Feng F., Yang G., Ma X., Zhang J., Huang C., Ma X., La Y., Yan P., Zhandui P., Liang C. (2024). Polymorphisms within the PRKG1 Gene of Gannan Yaks and Their Association with Milk Quality Characteristics. Foods.

[63] Liu L., Hao M., Zhang J., Chen Z., Zhou J., Wang C., Zhang H., Wang J. (2023). FSHR-mTOR-HIF1 Signaling Alleviates Mouse Follicles from AMPK-Induced Atresia. Cell Rep..

[64] Li Y.-T., Chen F.-Z., Chen W., Zhu H.-M., Chen Y., Li Z.-L., Yan F., Liu Z.-Y., Dong W.-R., Zhang L. (2022). Cdc42 Promotes Axonogenesis of Primary Hippocampal Neurons by Inhibiting Glycogen Synthase Kinase-3β. J. Integr. Neurosci..

[65] Mei Q., Li H., Liu Y., Wang X., Xiang W. (2022). Advances in the Study of CDC42 in the Female Reproductive System. J. Cell. Mol. Medi..

[66] Umbayev B., Saliev T., Safarova (Yantsen) Y., Yermekova A., Olzhayev F., Bulanin D., Tsoy A., Askarova S. (2023). The Role of Cdc42 in the Insulin and Leptin Pathways Contributing to the Development of Age-Related Obesity. Nutrients.

